# A prediction model for skeletal muscle evaluation and computed tomography-defined sarcopenia diagnosis in a predominantly overweight cohort of patients with head and neck cancer

**DOI:** 10.1007/s00405-022-07545-x

**Published:** 2022-07-14

**Authors:** Belinda Vangelov, Judith Bauer, Daniel Moses, Robert Smee

**Affiliations:** 1 Department of Radiation Oncology, Nelune Comprehensive Cancer Centre, Prince of Wales Hospital and Community Health Services, Level 1 | Bright Building | Barker St, Randwick, NSW 2031 Australia; 2 Prince of Wales Clinical School, Faculty of Medicine, University of New South Wales, Randwick, NSW 2031 Australia; 3 Department of Nutrition, Dietetics and Food, School of Clinical Sciences, Monash University, Clayton, VIC 3168 Australia; 4 Graduate School of Biomedical Engineering, University of New South Wales, Randwick, NSW 2031 Australia; 5 Department of Radiology, Prince of Wales Hospital and Community Health Services, Randwick, NSW 2031 Australia; 6 Department of Radiation Oncology, Tamworth Base Hospital, Tamworth, NSW 2340 Australia

**Keywords:** Head and neck cancer, Sarcopenia, Skeletal muscle, Computed tomography, Body composition

## Abstract

**Purpose:**

This study investigates the feasibility of computed tomography (CT)-defined sarcopenia assessment using a prediction model for estimating the cross-sectional area (CSA) of skeletal muscle (SM) in CT scans at the third lumbar vertebra (L3), using measures at the third cervical level (C3) in a predominantly overweight population with head and neck cancer (HNC).

**Methods:**

Analysis was conducted on adult patients with newly diagnosed HNC who had a diagnostic positron emission tomography–CT scan. CSA of SM in CT images was measured at L3 and C3 in each patient, and a predictive formula developed using fivefold cross-validation and linear regression modelling. Correlation and agreement between measured CSA at L3 and predicted values were evaluated using intraclass correlation coefficients (ICC) and Bland–Altman plot. The model’s ability to identify sarcopenia was investigated using Cohen’s Kappa (*k*).

**Results:**

A total of 109 patient scans were analysed, with 64% of the cohort being overweight or obese. The prediction model demonstrated high level of correlation between measured and predicted CSA measures (ICC 0.954, *r* = 0.916, *p* < 0.001), and skeletal muscle index (SMI) (ICC 0.939, *r* = 0.883, *p* < 0.001). Bland–Altman plot showed good agreement in SMI, with mean difference (bias) = 0.22% (SD 8.65, 95% CI − 3.35 to 3.79%), limits of agreement (− 16.74 to 17.17%). The model had a sensitivity of 80.0% and specificity of 85.0%, with moderate agreement on sarcopenia diagnosis (*k* = 0.565, *p* = 0.004).

**Conclusion:**

This model is effective in predicting lumbar SM CSA using measures at C3, and in identifying low SM in a predominately overweight group of patients with HNC.

## Introduction

Malnutrition is common in patients with cancer and is characterised by a decline in muscle mass, with, or without adipose tissue loss, due to a reduced nutritional intake, metabolic derangements, and/or toxicities of treatment modalities [1–3]. Sarcopenia is defined as low skeletal muscle mass and quality, a reduction in muscle strength, and subsequent decline in physical performance [3], and may or may not be evident in malnourished and/or visibly wasted patients [4]. Initially used to describe wasting and decline in function in the elderly population [5], sarcopenia is now recognised as a significant prognostic indicator in patients with various cancers [6], including head and neck cancer (HNC) [7–9]. Sarcopenia in HNC has been shown to be independently associated with reduced overall survival and may impact treatment-related toxicities [8, 9].

Much of the research conducted in oncology literature investigating sarcopenia opportunistically utilises routine diagnostic computed tomography (CT) scans, defining sarcopenia in terms of low muscle mass only, without function assessment, and is often termed “CT-defined” or “radiologically-defined” sarcopenia. Mention of sarcopenia in this paper will, therefore, refer to CT-defined sarcopenia. CT scans are readily available for retrospective studies in this population without an increased burden to patients, as scans are already conducted for staging purposes [10]. The assessment of skeletal muscle using CT scans has become the gold standard in body composition analysis at the tissue-organ level, with measurement of skeletal muscle in the cross-sectional area (CSA) of a single axial slice at the level of the third lumbar (L3) vertebra demonstrated to best correlate with whole body skeletal muscle [11–13]. In patients with HNC, however, whole body scans that include the L3 landmark are not always available, and this has led to the development of alternate options for skeletal muscle measurement at other vertebral levels, with varied results [14]. Since 2016, the most commonly used alternate vertebral landmark in patients with HNC has been the third cervical (C3) level, with at least two prediction equations developed to estimate the muscle area at L3 using measures taken at C3 [15, 16].

The prediction equation developed by Swartz et al. utilised the CSA measures at L3 and C3 in a cohort of 52 Dutch HNC patients [15], and has since been used to assess outcomes in numerous HNC populations [17–25] Jung et al. proposed an alternate model to estimate muscle area in 305 Korean HNC patients [16]. In both populations, the median body mass index (BMI) was within the healthy weight range (24.3 kg/m^2^ and 23 kg/m^2^, respectively). Our group has demonstrated previously that there may be issues in the translatability and accuracy of sarcopenia diagnosis, specifically with the application of the Swartz et al. [15] model, in differing populations, especially in those with a higher proportion of overweight or obese patients. We found weak agreement between measures in our predominantly overweight population [26].

The aim of the present study was to investigate the feasibility of a prediction model for estimating the muscle CSA at L3 (L3-CSA) using CSA at C3 (C3-CSA), that may be more suitable in a predominantly overweight population, and to investigate its reliability and applicability for sarcopenia assessment based on previously defined BMI and sex-specific thresholds [27].

## Materials and methods

### Study population and design

This is a single centre, ethics approved (2019/ETH13149), retrospective study of all adult (≥ 18 years) patients with a confirmed newly diagnosed squamous cell HNC, who presented to a tertiary referral Hospital in Sydney, Australia between January 2013 and June 2021.

All patients who presented with HNC of the larynx, hypopharynx, nasopharynx, oropharynx, or oral cavity, and had a baseline diagnostic positron emission tomography–computed tomography (PET–CT) scan were included. PET–CTs only were used as both the L3 and C3 landmarks are accessible for evaluation. Exclusion criteria were: patients who had a previous cancer diagnosis or had received any treatment (e.g., radiotherapy, chemotherapy or cancer-related surgery), or those who had incomplete or unclear PET–CT scans. Height and weight were recorded at the time of initial consult and collected from patient medical records, measured within 2 weeks of having the scan.

### PET–CT scan analysis

Patient diagnostic PET–CT scans were accessed via electronic medical records and anonymised prior to analysis. Skeletal muscle CSA at L3 and C3 was evaluated using Slice-O-matic Version 5.0 (Tomovision, Montreal, Canada). Muscle was delineated manually by a single, trained researcher (trained and certified in the Alberta protocol, with a < 2% interrater variation achieved) (BV), and supervised in vertebral landmarking by a Senior Radiologist (DM), and identified using standard Hounsfield Units (HU) of − 29 to + 150HU [28, 29]. Single CT scan images were landmarked at L3 and C3 using previously defined techniques [11, 15] and muscle CSA was recorded at both sites for analysis. Prediction models were developed to estimate L3-CSA using measures at C3 (described below in statistical analysis).

### Sarcopenia assessment

Patients were categorised based on BMI status (kg/m^2^) and defined as underweight (BMI < 20.0), healthy weight (BMI 20.0–24.99), overweight (BMI 25.0–29.9) or obese (BMI ≥ 30.0). The prediction equation was applied to C3 muscle attenuation values to provide estimated L3-CSA. To classify sarcopenia, CSA data were normalised for stature, and skeletal muscle index (SMI, cm^2^/m^2^) was calculated for each patient using both measured L3 and estimated L3 values. Patients were categorised as having sarcopenia or not, stratified by BMI category as previously defined by Martin et al. [27], with threshold values for females of SMI < 41 cm^2^/m^2^ and in males < 43 cm^2^/m^2^ (if underweight or healthy weight range) and < 53 cm^2^/m^2^ (if overweight or obese).

### Statistical analysis and model development

Descriptive statistics were presented as frequencies and percentages, with data presented as mean ± standard deviation (SD), or median (interquartile range, IQR) as appropriate. Normality was determined using the Shapiro–Wilk test.

Approximately 80% of the cohort was randomly selected for establishing the model and the remainder used for validation. Univariate and multivariate linear regression was applied to develop the prediction equation for muscle CSA at L3 with the independent covariates of age, sex, weight, height, tumour site, and C3-CSA, as well as the dependent variable L3-CSA included in the regression analysis. Variables that achieved a *p* value < 0.05 at the multivariate level were included in the final prediction model. The model was trained using the fivefold cross-validation method and resultant coefficients noted [30]. This method ensured all patients were included to train the model and mitigated model over-fitting. The mean squared error (MSE) was calculated on the application of each of the five models, and the mean of the five coefficients of each model was adopted as the final prediction equation. Testing of the final prediction model was conducted on the 20% independent validation sample. Intraclass correlation coefficients (ICC) were derived for each training model and the final model to assess degree of correlation and agreement between measures.

Patients were categorised as being sarcopenic or not (using the above mentioned thresholds) and sensitivity and specificity of the model’s ability to diagnose sarcopenia was determined. Cohen’s Kappa (*κ*) measurement of agreement was applied to compare both groups. A Bland–Altman plot was used to visually determine level of agreement of predicted measures of SMI. A priori of 5% was determined for SMI measures as a cutoff for clinically acceptable values. As there is currently no set limit in the literature, this was based on clinical judgement of acceptable difference and the use of additional nutritional parameters for determining interventions [31]. All statistical analysis was performed using SPSS Statistics software package, Version 26.0 (IBM, Armonk, NY).

## Results

There were 116 PET–CT scans (of 116 patients), of which seven were deemed ineligible. Three patients had unclear scans of the C3 landmark due to extensive neck curvature, three patients had incomplete scans that did not include the L3 landmark, and one patient had a late diagnosis that was not carcinoma. A total of 109 patient PET–CT scans were analysed that included both L3 and C3. The majority were male (85%) with a mean (SD) age of 61 (± 10.4) years. Most presented with squamous cell carcinoma of the oropharynx (69%) and T1 stage disease (37%). The median BMI of the cohort was 27 kg/m^2^, and 64% had a BMI ≥ 25 kg/m^2^. Detailed patient characteristics are shown in Table 1.

**Table 1:** Patient characteristics

	Whole cohort *n* = 109 (%)
Sex	
Male	93 (85)
Female	16 (15)
Age (years)	
Mean ± SD	61 ± 10.4
Range	33–85
Tumour site	
Larynx	10 (9)
Hypopharynx	2 (2)
Oropharynx	75 (69)
Nasopharynx	10 (9)
Oral cavity	5 (5)
Unknown primary	7 (6)
Staging*	
T-stage	
Tis	1 (1)
T1	41 (37)
T2	26 (24)
T3	19 (17)
T4	16 (15)
Tx	6 (6)
N-stage	
N0	13 (12)
N1	33 (30)
N2	57 (52)
N3	6 (6)
M-stage	
M0	105 (96)
M1	4 (4)
BMI (kg/m^2^)	
Median (IQR)	27 (6)
Overweight (BMI 25.0–29.9)	39 (36)
Obese (BMI ≥ 30.0)	31 (28)

*SD* standard deviation, *Tis* tumour insitu, *Tx* unknown primary, *BMI* body mass index, *IQR* interquartile range

*7th Ed UICC TNM classification of malignant tumours

The independent co-variates found to be statistically significant in the multivariate regression were; C3-CSA, age, sex and baseline weight. The coefficients in each training model were applied to predict L3-CSA using C3 measures in five sets of the data. The final prediction equation derived from the fivefold cross-validation method, taking the mean of the five (Table 2), and validated on the test sample (not used to train the model) was as follows:

L3-CSA = 124.838 + [1.881 × C3-CSA (cm^2^)] – [24.687 × sex] – age (years) + [0.472 × weight (kg)]

(For Sex use value of “1” for males and “2” for females).

**Table 2:** Prediction models for estimating L3-CSA from C3 measures

Model	Intercept	C3-CSA	Sex	Age	Weight	MSE
A	128.571	2.128	− 26.336	− 1.073	0.374	332.99
B	106.385	1.853	− 25.429	− 0.761	0.527	335.69
C	116.322	1.682	− 22.992	− 0.874	0.580	336.80
D	123.236	1.877	− 23.986	− 0.978	0.446	331.21
E	149.676	1.864	− 24.692	− 1.314	0.432	343.37
Mean	124.838	1.881	− 24.687	− 1.000	0.472	

Five-fold cross-validation used to determine models with multivariate linear regression. The prediction equation was developed as an average of the five:

L3-CSA (cm^2^) = 124.838 + [1.881 × C3-CSA (cm^2^)] – [24.687 × sex] – age (years) + [0.472 × Wt (kg)]

(For Sex use value of “1” for males and “2” for females)

**Fig. 1:**
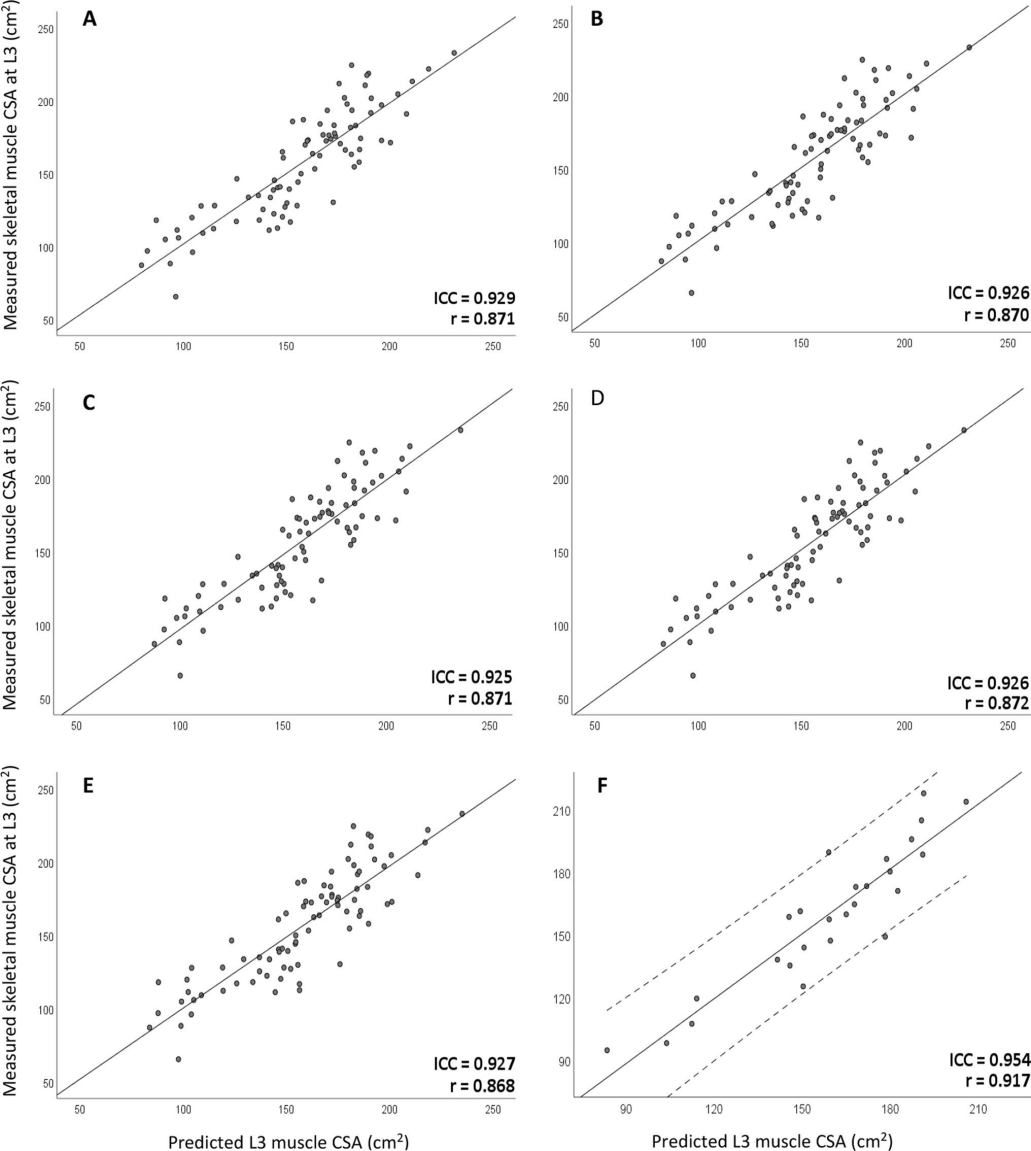
Scatterplots of measured L3-CSA and predicted L3-CSA of all five models in the cross-validation (**A**–**E**). Scatterplot F demonstrates application of final prediction model on test sample with confidence intervals in dotted lines. Intraclass correlation coefficients (ICC) included for each

The ICC values for each model are shown in Fig. 1 demonstrating that each training model had an ICC > 0.9 indicating excellent agreement of all training models, and the ICC of the final test model was 0.954 also indicating excellent agreement. Figure 1 also displays the distribution of measured L3-CSA values with the predicted values for CSA using C3 measures demonstrating a high level of correlation with measured L3-CSA when validated on the test sample (*r* = 0.917, *p* < 0.001). Strong correlation was also found when comparing predicted and measured SMI values (*r* = 0.883, *p* < 0.001). Four patients had missing height and, therefore, were not included in the sarcopenia analysis. At L3 in the test sample, 20% of patients were classified as sarcopenic, 28% using the prediction model.

**Fig. 2:**
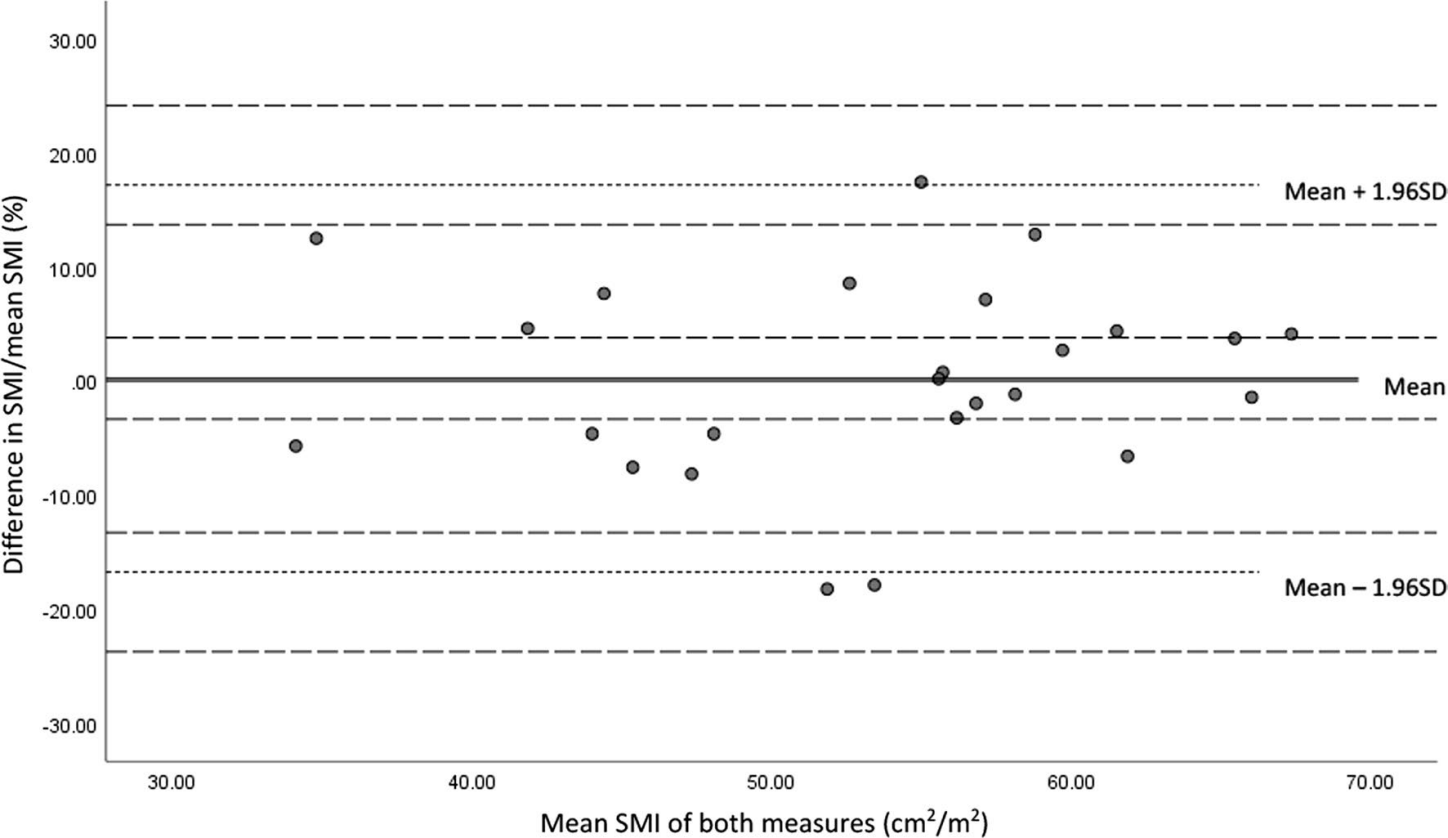
Bland–Altman plot of differences between measured L3 and predicted L3 values expressed as percentages of SMI against the mean of the measures, with limits of agreement (dotted lines) and 95% confidence intervals (dashed lines) in the test sample

Testing of the prediction model demonstrated a sensitivity of 80.0% and specificity of 85.0%, with moderate agreement on sarcopenia diagnosis (*k* = 0.565, *p* = 0.004). Bland–Altman plots demonstrated good agreement with mean difference (bias) in CSA measures = 0.72 cm^2^ (SD 13.45, 95%CI − 4.71 to 6.15 cm^2^), limits of agreement (LoA) (− 25.65 to 27.09 cm^2^). Good agreement was also found on SMI with a low level of proportional bias, mean difference (bias) = 0.22% (SD 8.65, 95% CI − 3.35 to 3.79%), LoA (− 16.74 to 17.17%), well under the set a priori of 5% in the test population (Fig. 2).

## Discussion

Sarcopenia in HNC patients is an independent prognostic indicator [8, 9], and early diagnosis is important to enable appropriate nutritional intervention planning. This study aimed to investigate the use of the vertebral landmark of C3 for muscle mass assessment in this population when L3 is not available in diagnostic CT scans. Although a prediction model has been previously suggested by Swartz et al. [15], our group has shown that equation to not have good agreement with gold standard measures at L3 in our population [26]. The present study is the first to our knowledge to explore the applicability of C3-CSA in prediction modelling for L3-CSA in a majority overweight or obese population. We propose an alternate prediction model using C3-CSA, which demonstrated strong agreement to L3 measures, and a higher specificity for sarcopenia diagnosis, tailored to our population.

Assessment of skeletal muscle at the level of L3 has become the gold standard in body composition measures at the tissue-organ level, and the most commonly used landmark [11] and is increasingly being used to diagnose sarcopenia in cancer patients. The use of alternate vertebral landmarks to L3 for CT-defined sarcopenia analysis has arisen due to the unavailability of diagnostic CT scans in patients with non-abdominal tumours, where L3 is visible. A recent systematic review identified researchers have used various levels from L1 to C3 in the pursuit of an alternative assessment method that can be applied to those without an abdominal scan [14]. The findings revealed mixed results, sarcopenia cut-off values that were not sex-specific, prediction models using heterogeneous and non-ethnicity specific populations, and that current evidence was not robust enough to suggest that an alternate vertebral level should replace L3 in skeletal muscle mass evaluation. The most frequently used prediction model in HNC is currently C3, with the equation to convert CSA measured at C3 into predicted values at L3 developed by Swartz et al. [15]. That model, however, was developed from a relatively small (*n* = 52), heterogeneous cohort of patients with HNC and cross-validation was not conducted. As previously mentioned, evaluation of the application of the model on an Australian population of mostly overweight or obese patients found that it had weak agreement with measures taken at L3 to an acceptable clinical level [26]. A mean percentage difference in SMI measures of 5.6% was found (range − 34 to 33.1%, *r* = 0.548, *p* < 0.001), which translated clinically to a 5% misclassification of sarcopenia. As a result, we developed a more applicable prediction model in the current study, as it seems that one equation may not “fit” all populations.

The independent variables used in this prediction model were determined using multivariate linear regression with those found to be statistically significant contributors included in the final equation. Age, weight at presentation, sex, and C3-CSA were all applied, and these were the same used in models proposed by Swartz et al. [15] and Jung et al. [16]. The variation in the corresponding coefficient values in each equation is indicative of the need for population based models, with one of the main differences in our population being a higher median BMI value (27 kg/m^2^ vs 24.3 kg/m^2^ and 23.0 kg/m^2^, respectively).

It is known that patients with HNC are at high risk of malnutrition [32, 33]. A recent position statement issued by the Clinical Oncological Society of Australia recommends screening all patients with cancer for both malnutrition and sarcopenia at the time of diagnosis [34]. As malnutrition in patients with HNC is a stronger prognostic predictor than CT-defined sarcopenia alone, assessment of both is crucial for intervention planning [35]. Determining those at highest risk of malnutrition at the time of diagnosis, however, can be challenging, especially with the increase in incidence of patients with human papillomavirus-related disease [36]. This particular group of patients often present as overweight or obese and may not display visible signs of wasting or nutritional issues, however, may be malnourished and/or sarcopenic, as these conditions are not exclusively associated with being underweight [37]. Without an alternative method to L3 muscle measurement, many patients with HNC, with sarcopenic obesity in particular, may go undiagnosed. The prediction model developed in this study provides a validated option in a predominantly overweight or obese population. The equation has a similar sensitivity but higher specificity for sarcopenia diagnosis (80.0% and 85.0%, compared to 79.2% and 66.7%, respectively), and a stronger agreement when compared to the most frequently used alternative [26].

The use of a high cervical vertebral landmark for skeletal muscle evaluation remains controversial, as it has yet to be determined whether the muscles in the neck can truly be used as a surrogate measure of abdominal muscle, which itself is a surrogate measure of whole body skeletal muscle, and if in fact both deplete at the same rate over time. It has been reported that the further away from L3 that measures are taken, the less they reflect whole body muscle mass [38]. Therefore, prediction models identifying sarcopenia should not be used without also including full nutritional assessments for appropriate intervention planning, as a patient may be malnourished and not necessarily sarcopenic and vice versa. Measures taken at this landmark should only be used for patient screening, or as an additional nutritional assessment tool, and not in isolation to full patient assessments. The better specificity of the model for our population indicates that there is not likely a “one equation for all”, and all prediction models should be validated based on specific populations, taking into account race, ethnicity, and sex, and applied in relevant cohorts. Future work in this area will likely involve artificial intelligence and the use of machine learning to allow automated muscle analysis, as manual delineation is time consuming and not always practical in the clinical setting. Many centres may not have access to such technology, however, and prediction modelling will likely be required for some time.

This study has several limitations, including its retrospective design and that not all patients presenting to the clinic had a PET–CT scan. The decision to have patients scanned, and the method used, is an Oncologist-specific preference in our facility, and as such, there may be bias in the selected cases. Some patients, for example, may just have a head and neck CT, and these patients were eliminated in this study due to the lack of L3 present for prediction modelling application. The use of the C3 level in HNC has its own limitations, as patients may have tumours that obscure accurate muscle identification, especially in the sternocleidomastoid muscles. This was not an issue with this cohort, as no patients had muscle-infiltrating tumours, and muscle delineation was possible for most, except those with significant spinal curvature. As is common in HNC, the majority of the cohort were male (85%) and this did not allow for testing of the model on larger numbers of female patients. The model, however, does allow for differentiation between sexes in the equation. Nevertheless, our cohort of over 100 patients, and the cross-validation method used (that included female patients in each validation set), ensured a prediction equation with good agreement. Larger studies with a higher proportion of females are likely needed to test this further; however, this would be difficult to achieve with the relatively lower numbers of female patients compared to males with HNC globally. The majority of patients had oropharyngeal carcinoma, and this may not be a representative sample of patients with HNC which might have some impact on results; however, the use of the fivefold cross-validation method allows for a robust prediction equation formulation.

## Conclusion

The prediction model developed in this study for the use of C3-CSA measures to estimate L3-CSA was found to have better agreement, and specificity than the Swartz et al. [15] equation in this Australian population. This suggests that our prediction equation may be more effective in recognising sarcopenic obesity, and be more suited to skeletal muscle evaluation in similar patient demographic cohorts, where it is used in conjunction with full nutritional assessment. Testing in larger populations is required.
